# Specific Alteration of Branched-Chain Amino Acid Profile in Polycystic Ovary Syndrome

**DOI:** 10.3390/biomedicines11010108

**Published:** 2023-01-01

**Authors:** Katarzyna Paczkowska, Dominik Rachoń, Andrzej Berg, Jacek Rybka, Katarzyna Kapczyńska, Marek Bolanowski, Jacek Daroszewski

**Affiliations:** 1 Department of Endocrinology, Diabetes and Isotope Therapy, Wroclaw Medical University, 50-367 Wrocław, Poland; 2 Department of Clinical and Experimental Endocrinology, Medical University of Gdansk, 80-211 Gdańsk, Poland; 3 Department of Pharmaceutical Chemistry, Medical University of Gdansk, 80-416 Gdańsk, Poland; 4 Laboratory of Medical Microbiology, Hirszfeld Institute of Immunology and Experimental Therapy, Polish Academy of Sciences, 53-114 Wrocław, Poland

**Keywords:** PCOS, BCAA, hyperandrogenemia, insulin resistance, abdominal obesity

## Abstract

Polycystic ovary syndrome (PCOS) is one of the most common endocrinopathies in reproductive age women; it is a complex health issue with numerous comorbidities. Attention has recently been drawn to amino acids as they are molecules essential to maintain homeostasis. The aim of the study was to investigate the branch chain amino acid (BCAA) profile in women with PCOS. A total of 326 women, 208 diagnosed with PCOS and 118 healthy controls, participated in the study; all the patients were between 18 and 40 years old. Anthropometrical, biochemical and hormonal parameters were assessed. Gas-liquid chromatography combined with tandem mass spectrometry was used to investigate BCAA levels. Statistical analysis showed significantly higher plasma levels of BCAAs (540.59 ± 97.23 nmol/mL vs. 501.09 ± 85.33 nmol/mL; *p* < 0.001) in women with PCOS. Significant correlations (*p* < 0.05) were found between BCAA and BMI, HOMA-IR, waist circumference and total testosterone levels. In the analysis of individuals with abdominal obesity, there were significant differences between PCOS and controls in BCAA (558.13 ± 100.51 vs. 514.22 ± 79.76 nmol/mL) and the concentrations of all the analyzed amino acids were higher in the PCOS patients. Hyperandrogenemia in PCOS patients was associated with significantly higher leucine, isoleucine and total BCAA levels. The increase of BCAA levels among PCOS patients in comparison to healthy controls might be an early sign of metabolic alteration and a predictive factor for other disturbances.

## 1. Introduction

Polycystic ovary syndrome (PCOS) is a one of the most common endocrinopathies in reproductive age women, affecting 6 to 20% of them according to different criteria [1,2], with a wide range of clinical manifestations, including menstrual irregularity, impaired fertility and cutaneous signs. However, PCOS is not only a reproductive disorder but also a complex health issue with numerous comorbidities; it is associated with a higher ratio of admissions to hospitals from various causes [3] and decreased work ability [4]. Several metabolic disturbances were found in PCOS, affecting carbohydrate, fat and protein metabolism [5]. Women with PCOS have an increased risk of insulin resistance (IR), type 2 diabetes, obesity and cardiovascular disease [3] and they are predisposed to develop metabolic syndrome with a rate of progression as high as 50% [6]. The mechanisms underlying the connection between PCOS and metabolic disorders are still not well understood [7]; however, PCOS patients might be considered as a biological model using research to study complex metabolic disturbances.

Insulin resistance is found in approximately 50–80% of PCOS individuals [8], and a connection between IR and hyperandrogenism is still being investigated. It was found that increased levels of insulin enhance the secretion of ovarian androgens [9] and inhibit the production of sex hormone-binding globulin [10]. Additionally, androgen excess seems to be correlated with the reduction of insulin sensitivity in skeletal muscles [11]. 

Attention has recently been drawn to amino acids (AAs), as they are molecules essential to maintain homeostasis [12]. Skeletal muscles account for approximately 40% of body mass and they are the most significant depository of free and bonded in the proteins amino acids in the body [13]. Amino acids are the precursors of polypeptides and proteins, but an increasing amount of evidence in the literature shows that they are regulating factors in a variety of metabolic pathways as well [14]. The link between glucose and amino acid metabolism is widely described; AAs might be the substrates for gluconeogenesis, but glucose can be a substrate for non-essential AA synthesis as well [15].

The amino acids involved in the regulation of key metabolic processes are called “functional AA” and this group includes leucine, tryptophan, glutamine, proline, cysteine and arginine [13]. Amino acids may be categorized by the chemical structure as well; such classification has been used in the present study, in which branched-chain AAs (BCAA) were analyzed.

Structurally similar BCAA groups consist of valine (VAL), leucine (LEU) and isoleucine (ILE) and they are included in the group of essential AAs that cannot be synthesized in the human body and must be provided with food [16]. Recently, BCAAs have been found to link several physiological processes, regardless of protein synthesis; [17] it was suggested that BCAAs participate in the regulation of glucose, lipid and protein metabolism [18] and also play a role in mitochondrial processes [19]. Leucine has been found to activate the mTOR—a metabolic pathway that connects nutrition with aging. [20,21] Several studies revealed higher levels of BCAA in patients with insulin resistance or type 2 diabetes mellitus and the correlation of BCAAs with HOMA-IR [22]. 

The alteration in the AA profile has been proposed as an early sign of developing metabolic disturbances. Simultaneously, PCOS patients develop numerous metabolic disorders at a younger age than other women. Taking into account those facts, PCOS individuals might be considered as the biological model of early metabolic disturbances. Therefore, in the present study, the BCAA profile in PCOS patients was analyzed to look for a link between AAs and anthropometrical, biochemical and hormonal parameters, including known markers of affected metabolism. 

## 2. Material and Methods

### 2.1. Study and Control Groups

A total of 326 women, 208 diagnosed with PCOS and 118 healthy controls, participated in the study. All patients were between 18 and 40 years old, and they had no history of diabetic or hypolipemic therapy. If they had taken hormonal contraceptives, the treatment had been discontinued at least 6 months before blood tests were performed. The control group had regular menses and normal ovarian morphology assessed in ultrasound examination. Polycystic ovary syndrome was diagnosed according to the revised 2003 Rotterdam criteria [23]. The study was approved by the Bioethics Committee of the Medical University of Gdańsk (permission number NKBBN/27/2018) and all the subjects gave written consent to participate.

### 2.2. Anthropometrical Parameters

Anthropometrical parameters such as weight, height and waist circumference were assessed with standard techniques. Body mass index (BMI) was calculated as: BMI = weight [kg]/height^2^ [m^2^]. 

Obesity was diagnosed in patients with BMI ≥ 30 in accordance with WHO criteria [24]. Applying the BMI criterium, for further analysis, the study population was categorized as obese (Ob+) or non-obese (Ob−).

Abdominal (central) obesity was defined as waist circumference (WC) greater or equal to 80 cm [25]. Therefore, in analysis, the subgroup of patients with abdominal obesity (AbO+) was separated and included 143 PCOS patients and 74 controls.

Body composition (percentage of fat, fat-free and muscle mass) was measured with bioelectrical impedance.

### 2.3. Biochemical and Hormonal Assessment

Blood samples for biochemical and hormonal assessment were collected after overnight fasting and measurements were performed using commercially available methods. 

Insulin resistance was assessed with homeostatic model assessment of insulin resistance (HOMA IR) and HOMA IR was calculated using the formula: HOMA IR = insulin (µU/mL) × glucose (mmol/L)/22.5. The upper range for HOMA IR was taken with a value of 2.5 [26,27,28]. Our study population was divided into groups, based on whether they had insulin resistance (IR+) or not (IR−), according to values of HOMA-IR. The IR+ group was classified as 85 PCOS women and 30 controls, while the IR− group was classified as 123 PCOS and 88 control.

Free androgen index (FAI) was calculated by the following formula: FAI = total testosterone [nmol/L] ∗ 100/SHBG [nmol/L] and reference interval 0.6–4.4 was applied as it was determined for immunoassay [29]. In the present study, hyperandrogenemia was diagnosed when level of total testosterone or androstenedione was above the upper laboratory range or FAI was greater than 4.4. Among PCOS patients, 121 of them were diagnosed with hyperandrogenemia (HA+) and 87 without (HA−).

### 2.4. Branched-Chain Amino Acids Profile Assessment

Gas-liquid chromatography combined with tandem mass spectrometry (GLC-MSMS Focus GC—IonTrap ITQ700 (Thermo) system) was used to assess AA levels. The methodology was consistent with the one described in a previous study [30].

### 2.5. Statistical Analysis

Statistical analyses were conducted using Statistica (TIBCO), version 13.3. Comparisons of anthropometrical, biochemical, hormonal and BCAA profiles were assessed using *t*-test and the Mann–Whitney U test for normally and non-normally dispersed parameters, respectively. The correlations were calculated with the Spearman correlation. *p* < 0.05 was taken as statistically significant in all analyses.

## 3. Results

A wide spectrum of anthropometrical parameters, endocrinological and biochemical blood test results, were compared between PCOS women and healthy women; these are presented in Table 1.

Several parameters were significantly higher in PCOS patients, including LH, LH/FSH ratio, prolactin, DHEA-S, testosterone, androstenedione, FAI, HOMA-IR and fasting insulin, while SHBG was lower in comparison to controls. The control group was accurately recruited and, apart from age and HOMA-IR, there were no significant differences between groups in the crucial metabolic parameters, especially BMI, waist circumference, lipid profile and percentage of body fat, fat-free and muscle mass, as well as in the level of the inflammatory marker—C-reactive protein (CRP).

Total BCAA concentrations were a differentiating factor between the PCOS and healthy individuals. Significant differences were observed both when BCAAs were analyzed as a group or separately. The results are presented in the Table 2. 

Biochemical, hormonal and anthropometrical parameters were analyzed as the potential factors that affect the BCAA profile. The Spearman coefficients are shown in Table 3; all the presented correlations were statistically significant. Positive correlations were found between BCAA and BMI, HOMA-IR, waist circumference, testosterone level, FAI and percentage of fat mass. Negative correlations were found between BCAA and estradiol, percentage of muscle and fat-free body mass.

The classification of the study population according to insulin resistance (IR+, IR−) showed significant differences in the IR+ subgroup between PCOS and control in total BCAA (579.82 ± 102.66 vs. 530.21 ± 105.22 nmol/mL, *p* < 0.05), as well as in the LEU, ILE and VAL concentrations when analyzed separately (respectively: 139.42 ± 23.8 vs. 128.4 ± 25.4 nmol/mL, *p* < 0.05; 83.5 ± 17.7 vs. 75.9 ± 17.2 nmol/mL, *p* < 0.05; 356.9 ± 67.2 vs. 325.9 ± 65.7 nmol/mL, *p* < 0.05). In this subpopulation, there were no significant differences in HOMA-IR between PCOS and control women (3.96 ± 1.36 vs. 3.52 ± 0.95; *p* = 0.12), while differences were observed in FAI, testosterone and androstenedione levels (respectively: 5.63 ± 4.09 vs. 2.50 ± 1.97, *p* < 0.001; 2.01 ± 0.77 vs. 1.04 ± 0.33 nmol/l, *p* < 0.001; 3.36 ± 1.34 vs. 2.01 ± 0.88 ng/mL, *p* < 0.001)

In the IR- subgroup, all the BCAA levels were higher in the PCOS group (total BCAA 513.47 ± 83.48 nmol/mL vs. 491.16 ± 75.56 nmol/mL; *p* = 0.05; VAL 313.14 ± 56.72 nmol/mL vs. 298.22 ± 50.65 nmol/mL; *p* = 0.07; LEU 126.59 ± 19.29 nmol/mL vs. 122.70 ± 17.53 nmol/mL; *p* = 0.12) but significant difference was only observed in the ILE levels (73.7 ± 12.9 vs. 70.2 ± 12.8 nmol/mL; *p* = 0.04).

The differences in AAs levels between PCOS and controls were further analyzed in the Ob+ and Ob− subgroups. The significant changes in amino acid profile continued to be observed in the non-obese subgroup, while in the obese subgroup there were no significant differences as is presented in Table 4.

The study population was subsequently divided into two subgroups with and without abdominal obesity (AbO+, AbO−). Total BCAA levels significantly differed between PCOS and the control group in the AbO+ subgroup (558.13 ± 100.51 vs. 514.22 ± 79.76 nmol/mL; *p* < 0.05) and concentrations of all the analyzed AAs were higher in PCOS patients (VAL 343.48 ± 65.69 vs. 314.29 ± 52.68 nmol/mL, *p* < 0.001; LEU 136.09 ± 22.83 vs. 126.41 ± 17.83 nmol/mL, *p* < 0.001; ILE 80.66 ± 16.26 vs. 72.79 ± 13.59 nmol/mL, *p* < 0.001). In the AbO- subgroup, the significant differences between PCOS and the control group were found only in the VAL level (311.67 ± 57.70 vs. 292.13 ± 57.30 nmol/mL *p* < 0.05). Total BCAA levels tended to be higher in PCOS patients but without statistical significance (509.02 ± 83.09 vs. 483.42 ± 92.37 nmol/mL; *p* = 0.053). It is worth mentioning that in the AbO+ subgroup, as in the whole study population, the waist circumference did not vary significantly (*p* = 0.20) between PCOS and the control group. However, the AbO+ PCOS women differed from healthy individuals in HOMA-IR (*p* < 0.001), FAI (*p* < 0.001), testosterone (*p* < 0.001) and androstenedione levels (*p* < 0.001).

The BCAA levels were analyzed in PCOS patients according to whether they had hyperandrogenemia (HA+, HA−), and the results are shown in Table 5. Hyperandrogenemia in PCOS patients was associated with significantly higher LEU, ILE and total BCAA. 

## 4. Discussion

Analyzing AA profiles in a large group of PCOS patients, major differences in comparison to healthy individuals were found. The most important finding was a significant increase in plasma BCAA, especially leucine, in PCOS patients with hyperandrogenemia. Additionally, more severe disturbances in BCAA were shown in PCOS patients when the analysis was made in a subgroup with abdominal obesity.

A significant difference in patients’ ages between the PCOS and control groups was observed in this study. In order to search for PCOS-specific differences related to BCAA metabolism, one of the basic conditions for the recruitment to the control group was a similarity with PCOS patients in most metabolic parameters already known as cardiovascular risk factors. Polycystic ovary syndrome individuals develop an altered metabolic profile at a younger age than healthy ones. Finally, there were no differences between those groups in LDL, TG, BMI, waist circumference, fasting glucose or body fat mass; however, significantly higher levels of fasting insulin and HOMA-IR were found. 

A difference in CRP levels between the groups was not observed in this study. In various research, a higher CRP level in PCOS women was explained by chronic inflammation due to metabolic alterations connected with an increased level of free radicals and oxidative stress [6]. Patients with PCOS are predisposed to more severe metabolic disturbances, which is why chronic inflammation is usually more strongly marked in this group [31]. A similarity in the CRP level between the groups suggests a comparable severity of metabolic derangements in the present study. Moreover, hormonal analysis did not show important differences apart from LH, testosterone and prolactin concentrations and the LH/FSH ratio, which is commonly known in PCOS women and has been confirmed in numerous studies [32,33].

The results from the present study showed significantly higher levels of BCAA in PCOS women compared with healthy ones, which is consistent with the previously published research [34,35,36,37,38], but the mechanism underlying this increase remains unknown. Although a high amount of protein in the diet and the over-nutrition of women with PCOS might be one of the causes, it was previously shown among men that the correlation of IR with a higher BCAA level did not arise from increased protein intake [39]. An increased BCAA concentration is considered the result of an altered catabolism due to the decreased activity of BCAA catabolic enzymes in adipose tissue [40,41]. 

While higher total BCAA concentrations in PCOS women were reported in several studies, the assessment of LEU levels in this group is inconsistent. In the current study, LEU concentration was significantly higher in PCOS women—both in the whole study group and in the subgroups with abdominal obesity or insulin resistance. Similar results have been presented in a few studies [34,35,41,42]. but opposite results have been published as well [43]. 

Leucine is one of the functional amino acids [14]; it may activate the mTOR pathway and by this mechanism enhance protein synthesis and, indirectly, also insulin signaling. [44] This process can be energetically costly, as it is usually connected with an increase in glucose uptake in tissues such as skeletal muscle [19]. What is more, LEU is suspected to stimulate insulin secretion from pancreatic b-cells [45] and to improve insulin sensitivity through various mechanisms. This potent insulin-sensitizing capability was shown in mice model research, in which leucine supplementation was given to one group and led to a reduction in HOMA-IR and the fasting insulin level [46].

The LEU function in metabolism regulation seems to not only be connected with insulin secretion and signaling. In vitro and animal studies revealed that LEU activates BCKDC, which limits the enzymes responsible for BCAA catabolism; it is speculated that LEU works as a nutritional signal that promotes BCAA disposal [47]. Our findings of LEU and the total BCAA concentration increase suggest a more complex regulation of BCAA clearance.

The results from the present study and the literature data lead us to the hypothesis that PCOS might be connected not only with IR but with resistance to different molecules involved in glucose homeostasis, including “leucine resistance”. A detailed explanation of this phenomenon is worth looking for to understand the mechanism of PCOS development; however, further studies are needed to assess if the alteration in LEU metabolism might be a part of PCOS pathogenesis or, rather, a secondary disfunction arising from impaired insulin signaling. 

An association of increased BCAA levels with the risk of IR regardless of obesity in normoglycemic middle-aged women [48] and in the PCOS population [34] was previously reported. It was suggested that BCAA excess might be a predictive factor of the development of IR [49,50] and type 2 diabetes mellitus [51,52], and it might promote IR by interference with insulin signaling in muscles [22]. 

In this study, BCAA concentrations were positively correlated with HOMA-IR. Among the subgroup of patients with insulin resistance, significant differences in BCAA concentrations were present between PCOS women and the control group, despite no differences in the IR parameters between those groups. These results support the hypothesis that metabolic disturbances, including alterations of the AA profile found in IR patients, are more severe in PCOS independently from the severity of IR. The underlying reasons for this PCOS-specific phenomenon should still be investigated.

The comparison of BCAA in the subpopulation of IR- PCOS patients revealed increased levels of all the BCAAs but only the ILE level was significantly changed, which is in contradiction to results from another study [34] They reported only an elevated level of VAL among PCOS women without IR. The inconsistency between studies might derive from differences in study populations, especially the ones in BMI and the severity of co-existing metabolic disturbances.

The present analysis revealed positive correlations of BCAA with BMI and the percentage of body fat mass. In the previous studies, significantly increased levels of circulating branched-chain amino acids were observed in obese patients and the BCAA levels were positively correlated with HOMA-IR [22]. The association of obesity and insulin resistance with BCAA was stronger than with lipid metabolites [22,53]. On the other hand, Chang et al. did not find a correlation between BCAA levels, BMI and the percentage of body fat mass [38]. The inconsistency might correspond to differences in the study design and the inclusivity criteria for the study cohort. The women from both the PCOS and control groups were older than our study subjects, and only overweight or obese women were included; that is why they presented a more severe metabolic phenotype. The difference between Asian and European populations in BMI impact on cardio-metabolic risk should also be kept in mind when comparing results from various studies.

Separating an Ob− subgroup revealed a similarity to the whole study population in an alteration of the BCAA profile between PCOS and control; however, differences were not observed in the Ob+ subgroup. This might support the hypothesis that an alteration in BCAA levels is an early sign of metabolic disturbances and distinguish lean PCOS patients and controls but the differences are less marked in obese individuals. This finding is inconsistent with another study, in which it was found that a higher BCAA level seems to distinguish PCOS women from women with metabolic syndrome [38].

As far as we know, we found for the first time an association of the BCAA profile with waist circumference. Abdominal obesity increases the incidence of cardiovascular disease risk factors in women with proper BMI [54] and it was proven in several studies that WC has a stronger association with other metabolic disturbances than BMI [55]. In the present study, central obesity, as a harm negative factor affecting the cardiovascular system, was correlated with BCAA alteration: a positive correlation of waist circumference with BCAA was found and the correlation was stronger than the one observed with BMI. In the AbO+ subgroup, concentrations of VAL, LEU and ILE were significantly increased in PCOS patients. In this subgroup, there was no difference in mean waist circumferences. However, the results might be affected by the differences in HOMA-IR to some extent.

Hyperandrogenemia is connected to IR and might be another factor that influences the AA profile but it has not yet been widely investigated in women. The literature data are very limited and the analysis of BCAA and hyperandrogenism in PCOS women was not discussed in most previous studies. The comparison of HA+ and HA− PCOS women in our study did not show significant differences in anthropometric parameters (BMI, waist circumference, percentage of body fat and muscle mass). Simultaneously, significantly increased LEU and ILE levels in HA+ in comparison to HA− PCOS women were observed. Moreover, BCAA levels were positively correlated with circulating total testosterone concentrations. The positive correlation of BCAA with testosterone was also observed in one study [37], but on the contrary no correlation was previously suggested. [38] In another analysis, androgen excess was connected to disturbances in lipid metabolism and not affecting LEU levels [34]. These inconsistences might result from a difference in hyperandrogenism definition; in our study only laboratory hyperandrogenemia was taken into account when patients were categorized as HA+ or HA−, while in other studies women with only clinical signs of hyperandrogenism were included in the HA+ group. 

## 5. Conclusions

Polycystic ovary syndrome is a heterogenous disorder that is also connected with changes in the serum BCAA profile. An increase in BCAA levels among PCOS women in comparison to healthy controls might be an early sign of metabolic alteration and a predictive factor for other disturbances. Moreover, an assessment of the influence of androgens on BCAA levels in the female population should be more closely investigated in future studies.

## 6. Limitations

The presented research also has some limitations. First of all, we were not able to assess the influence of diet and protein intake on the BCAA level. Additionally, body composition was assessed with bioelectrical impedance analysis, not with the gold standard of this measurement: dual-energy X-ray absorptiometry. Finally, it remains unclear whether an alteration in the BCAA profile is a part of pathogenesis or rather a result of other disturbances observed in PCOS women.

## Figures and Tables

**Table 1:** Analysis of anthropometrical, biochemical and hormonal parameters in PCOS and control groups.

	PCOS	Control	*p*
age	25.86 ± 5.38	31.08 ± 6.99	<0.001
BMI	26.09 ± 6.37	25.39 ± 5.22	0.67
waist circumference (cm)	89.75 ± 15.16	87.17 ± 14.59	0.12
fasting glucose (mg/dL)	87.22 ± 6.56	86.97 ± 8.37	0.41
HDL	65.69 ± 18.44	66.97 ± 15.64	0.40
triglycerides	95.39 ± 60.55	86.79 ± 42.61	0.31
total cholesterol	188.23 ± 35.60	188.89 ± 35.71	0.86
LDL	104.47 ± 33.37	104.46 ± 32.52	0.81
albumin	48.09 ± 2.85	47.35 ± 2.62	0.03
non-HDL	122.65 ± 37.94	121.91 ± 35.18	0.83
CRP	1.78 ± 3.28	1.72 ± 2.54	0.88
WBC	6.26 ± 1.57	5.67 ± 1.35	0.001
TSH	2.49 ± 1.55	2.10 ± 1.30	0.02
LH	9.57 ± 7.40	7.09 ± 6.03	<0.001
FSH	6.85 ± 4.05	6.76 ± 2.30	0.85
LH/FSH	1.46 ± 1.04	1.13 ± 0.98	<0.001
estradiol	232.79 ± 151.47	391.13 ± 179.84	<0.002
prolactin	433.11 ± 194.11	372.35 ± 152.38	0.006
DHEA-S	314.53 ± 125.92	204.22 ± 74.25	<0.001
testosterone	1.86 ± 0.67	1.05 ± 0.33	<0.001
SHBG	65.75 ± 38.41	76.77 ± 37.17	0.001
fasting insulin	11.83 ± 6.89	8.91 ± 4.80	<0.001
FAI	3.99 ± 3.29	1.75 ± 1.28	<0.001
androstendione	3.28 ± 1.31	2.13 ± 0.82	<0.001
HOMA-IR	2.55 ± 1.53	1.96 ± 1.15	<0.001
percentage of fat mass (%)	31.96 ± 10.41	30.32 ± 9.27	0.53
percentage of fat-free mass (%)	68.05 ± 10.41	69.68 ± 9.27	0.53
percentage of muscle mass (%)	64.95 ± 8.87	66.95 ± 8.83	0.23

BMI—Body Mass Index, CRP—C-reactive protein, WBC—white blood cells, TSH—thyroid-stimulating hormone, LH—luteinizing hormone, FSH—follicle-stimulating hormone, DHEA-S—dehydroepiandrosterone sulfate, SHBG—sex hormone-binding globulin, FAI—free androgen index, HOMA-IR—homeostatic model assessment of insulin resistance.

**Table 2:** Comparison of BCAA plasma concentration between PCOS and control group.

	PCOS	Control	*p*
VAL (nmol/mL)	331.02 ± 64.75	305.26 ± 55.90	<0.001
LEU (nmol/mL)	131.83 ± 22.12	124.15 ± 19.89	<0.001
ILE (nmol/mL)	77.73 ± 15.76	71.68 ± 14.20	<0.001
BCAA (nmol/mL)	540.59 ± 97.23	501.09 ± 85.33	<0.001

VAL—valine, LEU—leucine, ILE—isoleucine, BCAA—branched-chain amino acids.

**Table 3:** Correlation of BCAA level with selected parameters.

	BCAA
BMI	0.32
HOMA-IR	0.36
waist circumference	0.36
Estradiol	−0.25
Testosterone	0.20
FAI	0.34
percentage of fat-free mass	−0.37
percentage of fat mass	0.37
percentage of muscle mass	−0.39

BMI—Body Mass Index, HOMA-IR—homeostatic model assessment of insulin resistance, FAI—free androgen index.

**Table 4:** Comparison of BCAA profile between PCOS and control groups in the Ob− and OB+ subgroups.

	Non- Obese Individuals (Ob−)			Obese Individuals (Ob+)		
	PCOS	Control	*p*	PCOS	Control	*p*
VAL (nmol/mL)	321.21 ± 59.44	299.46 ± 54.38	0.003	365.59 ± 71.27	335.11 ± 53.77	0.06
LEU (nmol/mL)	128.42 ± 19.68	121.86 ± 19.91	0.002	143.86 ± 25.99	134.87 ± 16.79	0.10
ILE (nmol/mL)	75.14 ± 14.11	70.19 ± 13.36	0.002	86.86 ± 17.91	79.37 ± 14.54	0.12
BCAA (nmol/mL)	524.76 ± 87.69	491.51 ± 83.43	0.001	596.31 ± 109.08	549.34 ± 78.81	0.08

VAL—valine; LEU—leucine, ILE—isoleucine, BCAA—branched-chain amino acids.

**Table 5:** Differences in BCAA among PCOS patients with and without hyperandrogenemia.

	With Hyperandrogenemia (HA+)	Without Hyperandrogenemia (HA−)	*p*
VAL (nmol/mL)	338.54 ± 71.61	320.56 ± 52.41	0.10
LEU (nmol/mL)	136.47 ± 23.33	125.38 ± 18.59	<0.001
ILE (nmol/mL)	79.83 ± 16.89	74.81 ± 13.60	0.014
BCAA (nmol/mL)	554.85 ± 106.38	520.75 ± 79.24	0.02

VAL—valine; LEU—leucine, ILE—isoleucine, BCAA—branched-chain amino acids.

## Data Availability

The data presented in this study are available on request from the corresponding author. The data are not publicly available due to the ethical reasons.

## Abbreviations

AA—amino acid; AbO—abdominal obesity; BCAA—branched-chain amino acids; BMI—Body Mass Index; CRP—C-reactive protein; DHEA-S—dehydroepiandrosterone sulfate; FAI—free androgen index; FSH—follicle-stimulating hormone; HA—hyperandrogenemia; HOMA-IR—homeostatic model assessment of insulin resistance; ILE—isoleucine; IR—insulin resistance; LEU—leucine; LH—luteinizing hormone; Ob—obesity; PCOS—polycystic ovary syndrome; SHBG—sex hormone-binding globulin; TSH—thyroid-stimulating hormone; VAL—valine; WBC—white blood cells.

## References

[1] Yildiz B.O., Bozdag G., Yapici Z., Esinler I., Yarali H. (2012). Prevalence, phenotype and cardiometabolic risk of polycystic ovary syndrome under different diagnostic criteria. Hum. Reprod..

[2] Neven A.C.H., Laven J., Teede H.J., Boyle J.A. (2018). A Summary on Polycystic Ovary Syndrome: Diagnostic Criteria, Prevalence, Clinical Manifestations, and Management According to the Latest International Guidelines. Semin. Reprod. Med..

[3] Hart R., Doherty D.A. (2015). The potential implications of a PCOS diagnosis on a woman’s long-term health using data linkage. J. Clin. Endocrinol. Metab..

[4] Kujanpää L., Arffman R.K., Vaaramo E., Rossi H.R., Laitinen J., Morin-Papunen L., Tapanainen J., Ala-Mursula L., Piltonen T.T. (2022). Women with polycystic ovary syndrome have poorer work ability and higher disability retirement rate at midlife: A Northern Finland Birth Cohort 1966 study. Eur. J. Endocrinol..

[5] Murri M., Insenser M., Escobar-Morreale H.F. (2014). Metabolomics in polycystic ovary syndrome. Clin. Chim. Acta.

[6] Nawrocka-Rutkowska J., Szydłowska I., Jakubowska K., Olszewska M., Chlubek D., Szczuko M., Starczewski A. (2022). The Role of Oxidative Stress in the Risk of Cardiovascular Disease and Identification of Risk Factors Using AIP and Castelli Atherogenicity Indicators in Patients with PCOS. Biomedicines.

[7] Szmygin H., Lenart-Lipinska M., Szydelko J., Wozniak S., Matyjaszek-Matuszek B. (2022). Branched-chain amino acids as a novel biomarker of metabolic disturbances in women with polycystic ovary syndrome—Literature review. Ginekol. Pol..

[8] Legro R.S., Castracane V.D., Kauffman R.P. (2004). Detecting insulin resistance in polycystic ovary syndrome: Purposes and pitfalls. Obstet. Gynecol. Surv..

[9] Nestler J.E., Jakubowicz D.J., de Vargas A.F., Brik C., Quintero N., Medina F. (1998). Insulin stimulates testosterone biosynthesis by human thecal cells from women with polycystic ovary syndrome by activating its own receptor and using inositolglycan mediators as the signal transduction system. J. Clin. Endocrinol. Metab..

[10] Nestler J.E., Powers L.P., Matt D.W., Steingold K.A., Plymate S.R., Rittmaster R.S., Clore J.N., Blackard W.G. (1991). A direct effect of hyperinsulinemia on serum sex hormone-binding globulin levels in obese women with the polycystic ovary syndrome. J. Clin. Endocrinol. Metab..

[11] Rincon J., Holmäng A., Wahlström E.O., Lönnroth P., Björntorp P., Zierath J.R., Wallberg-Henriksson H. (1996). Mechanisms behind insulin resistance in rat skeletal muscle after oophorectomy and additional testosterone treatment. Diabetes.

[12] Siomkajło M., Daroszewski J. (2019). Branched chain amino acids: Passive biomarkers or the key to the pathogenesis of cardiometabolic diseases?. Adv. Clin. Exp. Med..

[13] Davis T.A., Fiorotto M.L. (2009). Regulation of muscle growth in neonates. Curr. Opin. Clin. Nutr. Metab. Care.

[14] Wu G. (2009). Amino acids: Metabolism, functions, and nutrition. Amino Acids.

[15] Gar C., Rottenkolber M., Prehn C., Adamski J., Seissler J., Lechner A. (2018). Serum and plasma amino acids as markers of prediabetes, insulin resistance, and incident diabetes. Crit. Rev. Clin. Lab. Sci..

[16] Neinast M., Murashige D., Arany Z. (2019). Branched Chain Amino Acids. Annu. Rev. Physiol..

[17] Hinkle J.S., Rivera C.N., Vaughan R.A. (2022). Branched-Chain Amino Acids and Mitochondrial Biogenesis: An Overview and Mechanistic Summary. Mol. Nutr. Food Res..

[18] Nie C., He T., Zhang W., Zhang G., Ma X. (2018). Branched Chain Amino Acids: Beyond Nutrition Metabolism. Int. J. Mol. Sci..

[19] Gannon N.P., Vaughan R.A. (2016). Leucine-induced anabolic-catabolism: Two sides of the same coin. Amino Acids.

[20] Anthony J.C., Anthony T.G., Kimball S.R., Jefferson L.S. (2001). Signaling pathways involved in translational control of protein synthesis in skeletal muscle by leucine. J. Nutr..

[21] Soultoukis G.A., Partridge L. (2016). Dietary Protein, Metabolism, and Aging. Annu. Rev. Biochem..

[22] Newgard C.B., An J., Bain J.R., Muehlbauer M.J., Stevens R.D., Lien L.F., Haqq A.M., Shah S.H., Arlotto M., Slentz C.A. (2009). A branched-chain amino acid-related metabolic signature that differentiates obese and lean humans and contributes to insulin resistance. Cell Metab..

[23] Rotterdam ESHRE/ASRM-Sponsored PCOS consensus workshop group (2004). Revised 2003 consensus on diagnostic criteria and long-term health risks related to polycystic ovary syndrome (PCOS). Hum. Reprod..

[24] World Health Organization (2000). Obesity: Preventing and managing the global epidemic. Report of a WHO consultation. World Health Organ. Tech. Rep. Ser..

[25] Alberti K.G., Zimmet P., Shaw J. (2006). Metabolic syndrome—A new world-wide definition. A Consensus Statement from the International Diabetes Federation. Diabet. Med..

[26] Stovall D.W., Bailey A.P., Pastore L.M. (2011). Assessment of insulin resistance and impaired glucose tolerance in lean women with polycystic ovary syndrome. J. Womens Health.

[27] Matthews D.R., Hosker J.P., Rudenski A.S., Naylor B.A., Treacher D.F., Turner R.C. (1985). Homeostasis model assessment: Insulin resistance and beta-cell function from fasting plasma glucose and insulin concentrations in man. Diabetologia.

[28] Jahromi B.N., Borzou N., Parsanezhad M.E., Anvar Z., Ghaemmaghami P., Sabetian S. (2021). Associations of insulin resistance, sex hormone-binding globulin, triglyceride, and hormonal profiles in polycystic ovary syndrome: A cross-sectional study. Int. J. Reprod. Biomed..

[29] Bui H.N., Sluss P.M., Hayes F.J., Blincko S., Knol D.L., Blankenstein M.A., Heijboer A.C. (2015). Testosterone, free testosterone, and free androgen index in women: Reference intervals, biological variation, and diagnostic value in polycystic ovary syndrome. Clin. Chim. Acta.

[30] Siomkajło M., Rybka J., Mierzchała-Pasierb M., Gamian A., Stankiewicz-Olczyk J., Bolanowski M., Daroszewski J. (2017). Specific plasma amino acid disturbances associated with metabolic syndrome. Endocrine.

[31] Guzelmeric K., Alkan N., Pirimoglu M., Unal O., Turan C. (2007). Chronic inflammation and elevated homocysteine levels are associated with increased body mass index in women with polycystic ovary syndrome. Gynecol. Endocrinol..

[32] Goudas V.T., Dumesic D.A. (1997). Polycystic ovary syndrome. Endocrinol. Metab. Clin. N. Am..

[33] Goodman N.F., Cobin R.H., Futterweit W., Glueck J.S., Legro R.S., Carmina E., American Association of Clinical Endocrinologists (AACE), American College of Endocrinology (ACE), Androgen Excess and PCOS Society (AES) (2015). American Association of Clinical Endocrinologists, American College of Endocrinology, and Androgen Excess and Pcos Society Disease State Clinical Review: Guide to the Best Practices in the Evaluation and Treatment of Polycystic Ovary Syndrome—Part 1. Endocr. Pract..

[34] Zhao Y., Fu L., Li R., Wang L.N., Yang Y., Liu N.N., Zhang C.M., Wang Y., Liu P., Tu B.B. (2012). Metabolic profiles characterizing different phenotypes of polycystic ovary syndrome: Plasma metabolomics analysis. BMC Med..

[35] RoyChoudhury S., Mishra B.P., Khan T., Chattopadhayay R., Lodh I., Datta Ray C., Bose G., Sarkar H.S., Srivastava S., Joshi M.V. (2016). Serum metabolomics of Indian women with polycystic ovary syndrome using ^1^H NMR coupled with a pattern recognition approach. Mol. Biosyst..

[36] Buszewska-Forajta M., Rachoń D., Stefaniak A., Wawrzyniak R., Konieczna A., Kowalewska A., Markuszewski M.J. (2019). Identification of the metabolic fingerprints in women with polycystic ovary syndrome using the multiplatform metabolomics technique. J. Steroid. Biochem. Mol. Biol..

[37] Ye Z., Zhang C., Wang S., Zhang Y., Li R., Zhao Y., Qiao J. (2022). Amino acid signatures in relation to polycystic ovary syndrome and increased risk of different metabolic disturbances. Reprod. Biomed. Online.

[38] Chang A.Y., Lalia A.Z., Jenkins G.D., Dutta T., Carter R.E., Singh R.J., Nair K.S. (2017). Combining a nontargeted and targeted metabolomics approach to identify metabolic pathways significantly altered in polycystic ovary syndrome. Metabolism.

[39] Tai E.S., Tan M.L., Stevens R.D., Low Y.L., Muehlbauer M.J., Goh D.L., Ilkayeva O.R., Wenner B.R., Bain J.R., Lee J.J. (2010). Insulin resistance is associated with a metabolic profile of altered protein metabolism in Chinese and Asian-Indian men. Diabetologia.

[40] Lackey D.E., Lynch C.J., Olson K.C., Mostaedi R., Ali M., Smith W.H., Karpe F., Humphreys S., Bedinger D.H., Dunn T.N. (2013). Regulation of adipose branched-chain amino acid catabolism enzyme expression and cross-adipose amino acid flux in human obesity. Am. J. Physiol. Endocrinol. Metab..

[41] Vanweert F., Schrauwen P., Phielix E. (2022). Role of branched-chain amino acid metabolism in the pathogenesis of obesity and type 2 diabetes-related metabolic disturbances BCAA metabolism in type 2 diabetes. Nutr. Diabetes.

[42] Whigham L.D., Butz D.E., Dashti H., Tonelli M., Johnson L.K., Cook M.E., Porter W.P., Eghbalnia H.R., Markley J.L., Lindheim S.R. (2014). Metabolic Evidence of Diminished Lipid Oxidation in Women With Polycystic Ovary Syndrome. Curr. Metab..

[43] Sun L., Hu W., Liu Q., Hao Q., Sun B., Zhang Q., Mao S., Qiao J., Yan X. (2012). Metabonomics reveals plasma metabolic changes and inflammatory marker in polycystic ovary syndrome patients. J. Proteome Res..

[44] Gannon N.P., Schnuck J.K., Vaughan R.A. (2018). BCAA Metabolism and Insulin Sensitivity—Dysregulated by Metabolic Status?. Mol. Nutr. Food Res..

[45] Newsholme P., Bender K., Kiely A., Brennan L. (2007). Amino acid metabolism, insulin secretion and diabetes. Biochem. Soc. Trans..

[46] Binder E., Bermúdez-Silva F.J., André C., Elie M., Romero-Zerbo S.Y., Leste-Lasserre T., Belluomo I., Duchampt A., Clark S., Aubert A. (2013). Leucine supplementation protects from insulin resistance by regulating adiposity levels. PLoS ONE.

[47] Harris R.A., Joshi M., Jeoung N.H. (2004). Mechanisms responsible for regulation of branched-chain amino acid catabolism. Biochem. Biophys. Res. Commun..

[48] Wiklund P., Zhang X., Pekkala S., Autio R., Kong L., Yang Y., Keinänen-Kiukaanniemi S., Alen M., Cheng S. (2016). Insulin resistance is associated with altered amino acid metabolism and adipose tissue dysfunction in normoglycemic women. Sci. Rep..

[49] Würtz P., Soininen P., Kangas A.J., Rönnemaa T., Lehtimäki T., Kähönen M., Viikari J.S., Raitakari O.T., Ala-Korpela M. (2013). Branched-chain and aromatic amino acids are predictors of insulin resistance in young adults. Diabetes Care.

[50] McCormack S.E., Shaham O., McCarthy M.A., Deik A.A., Wang T.J., Gerszten R.E., Clish C.B., Mootha V.K., Grinspoon S.K., Fleischman A. (2013). Circulating branched-chain amino acid concentrations are associated with obesity and future insulin resistance in children and adolescents. Pediatr. Obes..

[51] Wang T.J., Larson M.G., Vasan R.S., Cheng S., Rhee E.P., McCabe E., Lewis G.D., Fox C.S., Jacques P.F., Fernandez C. (2011). Metabolite profiles and the risk of developing diabetes. Nat. Med..

[52] Yamakado M., Nagao K., Imaizumi A., Tani M., Toda A., Tanaka T., Jinzu H., Miyano H., Yamamoto H., Daimon T. (2015). Plasma Free Amino Acid Profiles Predict Four-Year Risk of Developing Diabetes, Metabolic Syndrome, Dyslipidemia, and Hypertension in Japanese Population. Sci. Rep..

[53] Huffman K.M., Shah S.H., Stevens R.D., Bain J.R., Muehlbauer M., Slentz C.A., Tanner C.J., Kuchibhatla M., Houmard J.A., Newgard C.B. (2009). Relationships between circulating metabolic intermediates and insulin action in overweight to obese, inactive men and women. Diabetes Care.

[54] Shields M., Tremblay M.S., Connor Gorber S., Janssen I. (2012). Abdominal obesity and cardiovascular disease risk factors within body mass index categories. Health Rep..

[55] Lean M.E., Han T.S., Morrison C.E. (1995). Waist circumference as a measure for indicating need for weight management. BMJ.

